# A comprehensive research agenda for zero leprosy

**DOI:** 10.1186/s40249-020-00774-4

**Published:** 2020-11-12

**Authors:** Peter Steinmann, Courtenay Dusenbury, David Addiss, Fareed Mirza, W. Cairns S. Smith

**Affiliations:** 1 Swiss Tropical and Public Health Institute, Socinstrasse 57, 4051 Basel, Switzerland; 2 University of Basel, Basel, Switzerland; 3 Global Partnership for Zero Leprosy, Task Force for Global Health, Decatur, GA USA; 4 Focus Area for Compassion and Ethics, Task Force for Global Health, Decatur, GA USA; 5 Novartis Foundation, Basel, Switzerland; 6 University of Aberdeen, Aberdeen, UK

**Keywords:** Leprosy, *Mycobacterium leprae*, Priorities, Research, Strategy, Zero leprosy

## Abstract

**Background:**

Leprosy control achieved dramatic success in the 1980s–1990s with the implementation of short course multidrug therapy, which reduced the global prevalence of leprosy to less than 1 in 10 000 population. However, a period of relative stagnation in leprosy control followed this achievement, and only limited further declines in the global number of new cases reported have been achieved over the past decade.

**Main text:**

In 2016, major stakeholders called for the development of an innovative and comprehensive leprosy strategy aimed at reducing the incidence of leprosy, lowering the burden of disability and discrimination, and interrupting transmission. This led to the establishment of the Global Partnership for Zero Leprosy (GPZL) in 2018, with partners aligned around a shared Action Framework committed to achieving the WHO targets by 2030 through national leprosy program capacity-building, resource mobilisation and an enabling research agenda. GPZL convened over 140 experts from more than 20 countries to develop a research agenda to achieve zero leprosy. The result is a detailed research agenda focusing on diagnostics, mapping, digital technology and innovation, disability, epidemiological modelling and investment case, implementation research, stigma, post exposure prophylaxis and transmission, and vaccines. This research agenda is aligned with the research priorities identified by other stakeholders.

**Conclusions:**

Developing and achieving consensus on the research agenda for zero leprosy is a significant step forward for the leprosy community. In a next step, research programmes must be developed, with individual components of the research agenda requiring distinct expertise, varying in resource needs, and operating over different timescales. Moving toward zero leprosy now requires partner alignment and new investments at all stages of the research process, from discovery to implementation.

## Background

Following dramatic progress between 1980 and 2000, the control of leprosy (also known as Hansen’s disease), and reduction of the deep-rooted stigma and discrimination against people affected by the disease, have slowed considerably over recent years [1, 2]. In 2019, 202 185 new leprosy diagnoses were reported to the World Health Organization (WHO) [3]. The global implementation of multidrug therapy (MDT) for leprosy was a game-changer in the 1980s and 1990s [4]. The 1991 World Health Assembly (WHA) approval of a resolution to eliminate leprosy as a public health problem triggered the donation of MDT drugs to the WHO and innovations in leprosy case management and documentation, which led to a sharp reduction in the registered global prevalence of leprosy to less than 1 in 10 000 population [5]. Unfortunately, this strategy did not have a sustained impact on disease incidence; success and the perception that leprosy was no longer a problem led to a loss of political commitment to leprosy control, resulting in reduced resource allocation [4].

In September 2016, during the 19th International Leprosy Congress in Beijing, leading stakeholders called for the creation of a partnership to advance zero leprosy to achieve true elimination. Following extensive consultations, the Global Partnership for Zero Leprosy (GPZL) (https://zeroleprosy.org/) was formally established in 2018. It has over 700 members with a broad range of experience and expertise, and is managed by a leadership team and secretariat. GPZL’s vision of “no disease, no disability, and no discrimination/stigma” is in line with the targets and objectives of the WHO Global Leprosy Programme (https://www.searo.who.int/entity/global_leprosy_programme/en/), the International Federation of Anti-Leprosy Associations (ILEP; https://www.ilepfederation.org/), the Sasakawa Health Foundation, Novartis International and other key stakeholders. Members of the partnership have aligned around a shared Action Framework to achieve WHO’s 2030 global leprosy targets [6] through collaboration on country-led planning and capacity building, resource mobilization, and an enabling GPZL research agenda.

This Policy Platform describes the development, content and conclusions of the GPZL research agenda as well as the next steps in resourcing and implementing it. The leprosy community has diverse expertise and strong national and international dimensions, including in research, where the International Leprosy Association facilitates an International Leprosy Congress every three years. There are also national and regional leprosy associations, with a geographically-defined focus and often strong anchoring in local academic bodies. Global and local associations of people affected by leprosy, and non-governmental organisations working in leprosy, play an important role. Leprosy research is funded through grants from public and private bodies while national leprosy program directors and ministries of health are at the front line of defining implementation research needs. Consensus on any leprosy research agenda therefore has to involve all these stakeholders in order to be successful.

## Research agenda development

The GPZL Leadership Team appointed a chair in early 2018 to coordinate the development of the research agenda, with support from a senior expert advisor and the GPZL Secretariat. A working group was established with subgroup facilitators who led discussions across eight topic areas that had been selected by the Leadership Team members as priorities: (i) diagnostics; (ii) mapping, digital technology and innovation; (iii) disability; (iv) epidemiological modelling and investment case; (v) implementation research; (vi) stigma; (vii) post-exposure prophylaxis (PEP) and transmission; and (viii) vaccines. An open call for participants was issued to recruit experts and persons who had experienced leprosy. The research agenda process strived to engage the diversity of the leprosy community as well as the broader neglected tropical diseases (NTD) community to ensure agreement and ownership of the research priorities. Overall, more than 144 persons from over 20 countries signed up to participate.

A mapping of past initiatives to define leprosy research priorities, including those conducted by the Leprosy Research Initiative (LRI; https://www.leprosyresearch.org/) and the Research to Stop Neglected Tropical Diseases Transmission Initiative (R2STOP; https://r2stop.org/) provided a starting point. It was considered important that the research agenda be built on previous work and followed established principles for qualitative research (COREQ) [7]. The team also engaged with the NTD community through a panel discussion at the 2018 Coalition for Operational Research on NTDs (COR-NTD; https://www.ntdsupport.org/cor-ntd) meeting in New Orleans, USA. The detailed research priorities for each of the 8 priority themes was published in Leprosy Review [8] along with a commentary [9]. The current article focuses on the context of the research priority identification and their significance.

## Research areas and priorities

Similar to many other NTDs, leprosy is a complex condition in terms of its clinical and epidemiological characteristics, long-term medical and biological consequences (or effects), and intersections with socio-economic and cultural factors [10, 11]. Several cross-cutting themes emerged during the working group and subgroup deliberations, including the need for integration between research efforts. To achieve breakthroughs, research projects may need to integrate multiple disciplines and collaborate across traditionally separated fields. For example, research to understand the successful implementation of post-exposure prophylaxis (PEP) would necessarily need a complementary component on acceptability and the impact of stigma and discrimination on care-seeking behaviours.

Research should be prioritized based upon its potential impact and likelihood of leading to transformative, effective, and efficient innovations. At the same time, operational research that leads to stronger programmatic capacity and informs integration with, and strengthening of, national public health and health systems is needed to ensure that these new technical innovations are accessible and scalable at the national and sub-national levels. The need for high-quality leprosy research studies meeting the standards required for inclusion in WHO Guidelines has been noted [12].

A major partner in developing the research agenda was LRI, which was launched in 2013. The LRI conducted a similar priority-setting process in 2018 to inform its investment priorities. LRI adopted an elegant mixed-methods approach to defining and evaluating research priorities [13]. A recent editorial [9] compared the outcomes of the GPZL and LRI approaches and showed that, while the outcomes were not identical they were well aligned, offering welcome validation of the findings and reassuring the GPZL that its process has produced a robust research agenda.

The eight finalized research areas, and the priorities identified for each research area, are summarised in the panel.

As with other NTDs, COVID-19 poses significant challenges to leprosy control. WHO has issued interim guidance for community-based programs in the context of the COVID-19 pandemic [14], and national programs are restarting program interventions while relevant research gradually resumes, as the situation allows. Of particular concern are delays in diagnosis and interruption of treatment that might translate into increased morbidity, incomplete cure and the spread of drug resistance.

## Panel: the research priorities to achieve zero leprosy

### Diagnostics

The development, standardization, and deployment of accurate diagnostic tests for the early detection of infection and disease is a top priority. This may include molecular-based [15, 16] and immunological tools [17] that require digital support. Because a diagnosis of leprosy is usually made on clinical signs and symptoms and there is no “gold standard” nor easy method to correlate infection to disease progression, patients are often diagnosed and treated late, increasing the likelihood of further transmission and disease-related disability. Contact screening may offer opportunities for early diagnosis [18, 19] and targeted interventions. Another priority is harmonization, i.e., validation and quality assurance programmes to ensure standard procedures, correct interpretation, and thus high confidence in test results [20].

### Digital technology and innovation

Governments, policymakers, and other stakeholders are seeking scalable and sustainable digital health solutions that can be integrated into national health systems and, ideally, expanded to include other conditions [21]. Digital interventions such as eLearning, digital diagnostics, and geolocation of leprosy patients are priorities. A number of applications are under development, including digital registries; a leprosy referral and surveillance network among healthcare providers; tele-dermatology to support health workers with access to medical specialists [22]; and, smartphone apps to facilitate diagnosis and treatment for peripheral health workers [23]. Policy to support the practical implementation of these developments will also be needed.

### Disability

The early detection and treatment of leprosy is critical to preventing disabilities [24]. Effective strategies for preventing and stopping disability exist and rehabilitation techniques are available [25], but more knowledge to inform the accessibility, effectiveness, and cost-effectiveness of services is needed, as are new tools to improve practice. Better understanding of the causes of disability and ways to optimize disease management, is required for marginalised and economically and socially poor communities. Evidence on the significance of early detection [24], including the impact of case-finding and contact-tracing strategies on the prevalence of leprosy-related disabilities among new cases, will support morbidity prevention, as will work to better understand the pathophysiology, detection, and management of reactions. Minimizing the impact of living with impairments due to leprosy is another priority and requires studies on the prevention and treatment of disability and on the efficacy, accessibility, and effectiveness of rehabilitation services, assistive devices, and community-based rehabilitation programs [26]. Better estimates on the number of people disabled from leprosy (and their needs) and estimates of the burden of leprosy disability are necessary to understand and quantify the need and to allocate resources [27].

### Epidemiological modelling and investment case

Decisions on the selection and implementation of leprosy interventions [28, 29] should be based on a robust analysis of the benefits, risks, and costs [30, 31]. These include a financial and cost analysis of leprosy and an estimate of its socioeconomic burden. Currently, such evidence is scarce. Transmission models should be improved, as epidemiologic modelling is a powerful tool to prioritize alternative tools and interventions and evaluate endgame strategies as donors commit to zero leprosy [32]. An Elimination Investment Case (EIC) provides a framework for a systematic assessment of what is needed to achieve zero leprosy and the challenges, risk, and sustainability of various initiatives [33, 34].

### Implementation research

Implementation research is required to improve the functioning of national leprosy programs and to increase the effectiveness of collaborations with their long-term partners [35]. It is equally relevant to increasing the quality of leprosy data within countries and globally [36]. Priority operational issues include case mapping, data management, monitoring and surveillance, health systems strengthening, genetic and clinical drug resistance surveillance, and active case finding. Advances in mapping technologies such as global positioning systems and geographic information systems that combine discrete location data with mobile or static services [37, 38] have not yet been fully integrated by the leprosy community into routine operations to target interventions [39]. Better data are needed for decision-making [40]. The AIM Initiative (https://aiminitiative.org/) promotes integrated mapping of routine NTD data for evidence-based intervention planning. These operational research priorities can be pursued as individual topics or integrated into program evaluation targets, larger proposals and global health systems strengthening initiatives.

### Stigma

Among the targets in ILEP’s Triple Zero Campaign—Zero Transmission, Zero Disability, and Zero Discrimination—the third (which includes stigma and mental well-being) has received the least attention. This is despite stigma and attitudinal barriers being cited as major challenges by persons affected by leprosy [41]. Stigma is a barrier to zero leprosy in terms of missed prevention opportunities and access to treatment and appropriate case management, and improved mental well-being is central to reducing the burden of leprosy [42]. Interventions to reduce stigma such as support groups offering peer counselling, peer-to-peer networks led by local experts, socioeconomic development, and the involvement of persons affected in leprosy services have been studied but little standardization has been achieved [43]. Such interventions need to be validated in different settings [44, 45] and scale up must be explored in order to define standardized approaches.

### Post exposure prophylaxis (PEP) and transmission

Evidence for the efficacy of PEP with single dose rifampicin (SDR) has been established through multiple studies [46–49]. Among current research, the Leprosy Post-Exposure Prophylaxis (LPEP) program focuses on feasibility and impact [50–52]. Issues related to acceptability, perception [53], drug resistance [54] and impact of the treatment among those at highest risk of disease remain to be studied. The PEOPLE trial evaluates different PEP regimens and delivery modalities; the PEP4LEP trial assesses multiple contact tracing and screening platforms; the MALTALEP trial [55] examined the benefits of immunization with Bacille Calmette-Guerin (BCG) alone or in combination with SDR; and the PEP++ study [56] aims to establish an enhanced chemoprophylaxis regimen for close contacts of persons with leprosy. Another unmet research question pertains to the effect of chemoprophylaxis on transmission.

Priorities for understanding *M. leprae* transmission are related to understanding human-to-human transmission [57]. One of the main challenges to interrupting transmission is the long incubation period, during which transmission to contacts is assumed to occur. Other transmission-related research priorities are transmission networks, the extent and epidemiological significance of non-human reservoirs, and host–pathogen interactions. A better understanding of these might facilitate the development of diagnostic tests for both infection as well as pre-clinical and clinical disease [58].

### Vaccines

Until recently, immunotherapy options for leprosy were limited to the live vaccine BCG [59–61]. Renewed efforts to develop partially effective vaccines, such as different BCG strains, into improved leprosy vaccines [62, 63] have resulted in *Mycobacterium indicus pranii* (MIP), a whole cell vaccine of heat-killed mycobacteria [64]. The ideal vaccine against leprosy would need to induce strong, long-lasting T cell responses directed against *M. leprae* antigens, thereby limiting infection, preventing disease, and reducing bacterial transmission to others [65, 66]. Only recently has it been practical to contemplate the development and delivery of a new generation of leprosy vaccines. Of critical relevance for such vaccines is the recent availability of adjuvants that enable a new generation of T cell vaccines. LepVax is a multivalent recombinant protein formulated in a modern adjuvant that is used in more than a dozen vaccine candidates and is a safe and effective inducer of durable T cell responses [67]. It has been suggested that LepVax might first be used as a curative rather than a prophylactic vaccine [67]. For both MIP and LepVax, however, full clinical trials and registration in multiple countries have yet to be achieved and safety monitoring must be established. In the case of curative vaccines, sensitive diagnostic tests are critical.

## Conclusions

Despite the impact of MDT on leprosy, particularly in the 1990s, the number of reported cases remained stagnant over the last decade and concerns are growing that a significant number of cases may go unreported and undiagnosed [2, 68]. In addition to traditional public health approaches such as active case finding [69], new techniques and innovations are needed: these require innovative, high-quality research and an engaged scientific community that is aligned and committed to addressing key research priorities [70].

The recent WHO Leprosy Guidelines highlighted the insufficient quality of leprosy research in many areas [71]. The establishment of the GPZL, a coalition of all key actors, in 2018 has already revitalized thinking towards leprosy control through extensive engagement with leprosy stakeholders and the NTD community as a whole. Experience with elimination efforts for other NTDs has highlighted the importance of this type of commitment and alignment [72, 73]. For example, a comprehensive research agenda for the elimination of lymphatic filariasis (LF), commissioned by the Bill & Melinda Gates Foundation in 2004 under the auspices of the Global Alliance to Eliminate Lymphatic Filariasis (GAELF), catalysed key scientific research, resulted in the development of new tools and strategies, and focused funding for LF elimination around priority strategies [74].

For LF and a few other NTDs, including schistosomiasis, a research agenda development process and research priorities were commissioned and funded almost in their entirety by a single donor, namely the Bill & Melinda Gates Foundation. This has not been the case for leprosy. Thus, the challenge now is to mobilise resources to implement this research agenda, which will require further priority-setting and coordination on protocol development, the cooperation of experts and institutions with a wide range of expertise, availability of field sites, and very considerable financial investment.

The individual components of the research agenda vary in resource needs, will require distinct expertise, and will operate over different timescales. The stigma, disability, and PEP research plans are well advanced, with many activities in progress or even near implementation and scaling up. Epidemiologic modelling is being funded by the GPZL as part of its advocacy and resource mobilisation strategy in 2020. The operational research and digital health plans can be tackled and progress made in the short term, while innovations in the fields of diagnostics and vaccines will require much longer timescales.

The research agenda needs to be imaginatively yet loosely managed as there are considerable synergies between the different elements, such as PEP, vaccines, and diagnostics, yet research is notoriously unpredictable and progress cannot be fully controlled. Of particular importance is early input from stigma and disability experts in the development of tools and approaches that might touch on socially sensitive areas including concerns over disclosure and surveillance.

Developing and achieving consensus on the research agenda for zero leprosy is an important step forward for the leprosy community. The next step—further prioritization, partner alignment, resource mobilisation, planning, and coordination of the realisation of that research agenda—is equally critical. Moving towards zero leprosy requires investments from existing and new partners at all stages of the process, from discovery to implementation. Technical innovation is required to create the necessary tools for intervention and diagnosis. High-quality implementation research is needed to standardize those tools and bring them into national programs, supported by a strong evidence base. The G-FINDER report on research and development funding for NTDs including leprosy presents the scale of the current investment and the sources of funding [75]. However coordination is needed to ensure that resources are directed, in an impactful and measurable way, to the sustained, effective programs required to achieve zero leprosy. Ideally, coordination and control will increasingly be by the countries and communities most in need of innovations.

## Data Availability

From the GPZL.

## Abbreviations

BCG: Bacille Calmette-Guerin
GPZL: Global partnership for zero leprosy
LRI: Leprosy research initiative
MDT: Multi-drug therapy
MIP: *Mycobacterium indicus pranii*
NTDs: Neglected tropical diseases
PEP: Post-exposure prophylaxis
WHO: World Health Organization
